# Genetic testing for young-onset colorectal cancer: case report and evidence-based clinical guidelines

**DOI:** 10.2478/v10019-010-0005-0

**Published:** 2010-03-18

**Authors:** Yaolin Zhou, Lisa A. Boardman, Robert C. Miller

**Affiliations:** 1 Mayo Medical School, College of Medicine, Mayo Clinic, Rochester, Minnesota; 2 Division of Gastroenterology and Hepatology, Mayo Clinic, Rochester, Minnesota; 3 Department of Radiation Oncology, Mayo Clinic, Rochester, Minnesota

**Keywords:** adenomatous polyposis coli, attenuated familial adenomatous polyposis, colorectal cancer, familial adenomatous polyposis, microsatellite instability, *MYH*-associated polyposis

## Abstract

**Background:**

Young-onset colorectal cancer is clinicopathologically different from older-onset colorectal cancer and tends to occur in patients with hereditary germline conditions such as Lynch syndrome and familial adenomatous polyposis.

**Case report.:**

We describe the case of a 44-year-old man with a paternal history of colon polyps, a personal 2-year history of hematochezia, and a diagnosis of rectal cancer. Further clinical evaluation of the patient at our institution determined the cancer to be stage IIIA. The patient underwent genetic counseling and testing, which indicated he was negative for the most common familial cancer syndromes. After treatment with neoadjuvant chemoradiotherapy, surgery, and adjuvant chemotherapy, the patient has done well. We review the hereditary cancer syndromes and genetic tests to consider for patients with early-onset colorectal cancer.

**Conclusions:**

This case underscores the importance of following cancer-screening guidelines.

## Introduction

Colorectal cancer (CRC) is a common malignancy in North America and Europe; most patients have sporadic disease without a known genetic predisposition to the illness. With the exception of the hereditary germline conditions Lynch syndrome, familial adenomatous polyposis (FAP), and attenuated FAP, which are marked by an early age of onset and familial CRC clustering, most cases of CRC do not develop until age 65 years or older. Still, up to 20% of all cases of CRC arise in persons aged 50 years or younger who do not have Lynch syndrome or FAP.^1^–^3^

Lynch syndrome, formerly known as hereditary nonpolyposis colorectal cancer, is caused by a germline mutation in 1 of several DNA mismatch repair (MMR) genes and is the most common single-gene–related cause of hereditary colon cancer in North American and European populations.^4^,^5^ CRC in Lynch syndrome has unique histopathologic and clinical findings. These cancers tend to be more responsive to treatment despite being poorly differentiated.^2^

One report based on the experience from 2 cancer registries in the United States, the National Program of Cancer Registries and the Surveillance, Epidemiology and End Results studies, emphasized that CRC is of concern for young adults: it is among the top 10 cancers in persons aged 20 to 49 years of all races.^6^ Young-onset and older-onset CRC are clinicopathologically different in that young-onset CRC usually presents at a later stage and is more poorly differentiated.^6^ Here, we present the case of a young middle-aged man with rectal cancer.

## Case report

A 44-year-old man sought medical care in 2001 for a 2-year history of hematochezia. Colonoscopy performed at an outside institution in September 2001 showed a lesion within the rectum. Biopsy performed at that time confirmed a poorly differentiated, grade 3 adenocarcinoma, which measured 2×1.3×1 cm, with invasion through the muscularis mucosa. He came to our institution in October 2001 for further evaluation.

The patient’s family history of cancer was primarily limited to a brother who received a diagnosis of chronic myelogenous leukemia at age 47 years. His father had colon polyps removed in his mid 50s, and a maternal aunt had colon polyps at age 35 years. Distant paternal relatives may have had CRC in old age.

The patient had been a smoker for the previous 16 years and had a history of gastroesophageal reflux disease. He had a history of sebaceous cysts on the left scapula and the back of the thighs and a lipoma on the inferior border of the right scapula. He did not have supernumerary teeth.

**FIGURE 1:**
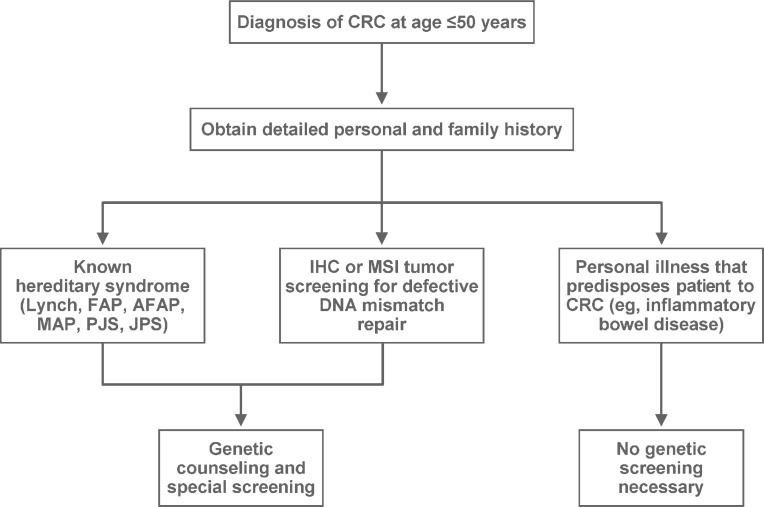
Scheme describing the recommended initial evaluation of a patient aged 50 years or younger with a diagnosis of colorectal cancer (CRC). AFAP indicates attenuated familial adenomatous polyposis; FAP, familial adenomatous polyposis; IHC, immunohistochemistry; JPS, juvenile polyposis syndrome; MAP, *MYH*-associated adenomatous polyposis; MSI, microsatellite instability; PJS, Peutz-Jeghers syndrome.

**FIGURE 2:**
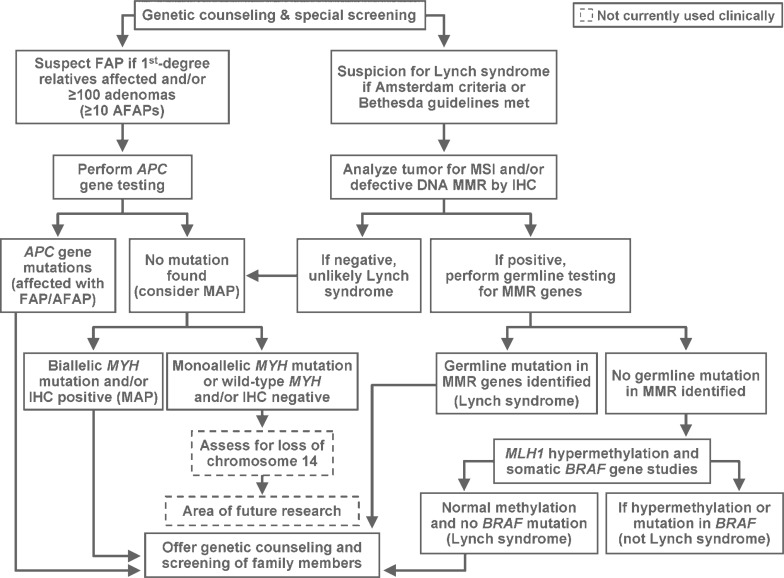
Scheme describing the recommended genetic testing for a patient with a diagnosis of colorectal cancer. AFAP indicates attenuated familial adenomatous polyposis; *APC*, adenomatous polyposis coli; *BRAF*, v-raf murine sarcoma viral oncogene homolog B1; FAP, familial adenomatous polyposis; IHC, immunohistochemistry; MAP, *MYH*-associated adenomatous polyposis; *MLH1*, MutL homolog 1; MMR, mismatch repair; MSI, microsatellite instability; *MYH*, MutY homolog.

Evaluation of the patient included genetics counseling and testing because of his young age at diagnosis (Figure 1). Immunohistochemical testing (IHC) for MLH1, MSH2, and MSH6 showed intact MMR. The patient was not tested for the *APC* gene mutation because he was not suspected to have FAP (Figure 2). He was also not tested for *MYH*, which was not discovered until later.^7^

Endorectal ultrasonography with guided fine-needle aspiration biopsy of suspicious lymph nodes confirmed an ulcerated lesion extending from the anal verge to 3.5 cm proximally along the anterior wall of the rectum. Fine-needle aspirates from 2 enlarged perirectal lymph nodes were positive for adenocarcinoma. In the ascending colon, a 5-mm hyperplastic polyp was removed with cold snare.

Abdominal and pelvic computed tomography detected a 0.6-cm sclerotic lesion within the left iliac bone, which was considered benign after a bone scan demonstrated no bony metastases. Results of upper endoscopy were normal. On the basis of the clinical evaluations, the patient’s cancer was determined to be stage IIIA (T2N1M0) by the American Joint Committee on Cancer 6th edition criteria.

Neoadjuvant chemoradiotherapy was recommended on the basis of the tumor stage and the proximity of the tumor to the prostate. From November through December 2001, the patient was given a continuous infusion of radiosensitizing 5-fluorouracil (5-FU) (225 mg/m^2^). He was treated with 180 cGy in 28 fractions, for a total of 5040 cGy. He tolerated the therapy well, with some mild diarrhea, weight loss, decreased energy, and perianal irritation. The cancer in the rectum showed complete response to therapy.

In January 2002, the patient underwent surgery to have an abdominoperineal resection and permanent colostomy. He tolerated the procedure well, and no residual tumor was identified. Two of 31 regional lymph nodes were positive for grade 3 mucinous adenocarcinoma. In the following month, the patient had several perineal drain site infections and was treated for depression.

In April 2002 the patient began his first of 4 cycles of adjuvant 5-FU and leucovorin systemic chemotherapy. Prolonged neutropenia and gastrointestinal tract bleeding occurred in the middle of the first cycle of chemotherapy; therefore, the dosage of 5-FU (425 mg/m^2^) was decreased by 10% (to 380 mg/m^2^). He completed chemotherapy in August 2002.

Surveillance colonoscopies in the following years have been negative. The patient was last seen at our institution in January 2007. He has had no new gastrointestinal tract or genitourinary symptoms or problems. His appetite has returned to normal, and he feels strong. He has returned to work as a carpenter and is now being monitored with colonoscopy every 3 years.

## Discussion

According to the Fearon and Vogelstein model of carcinogenesis, the accumulation of multiple mutations is required for the transformation of normal colonic mucosa into dysplastic adenomas and then into invasive carcinomas.^4^ CRC can be classified broadly as exhibiting either chromosomal instability through gain-of-function mutations (APC/β-catenin pathway) or microsatellite instability (MSI) (defects in DNA MMR). In sporadic CRC, mutations are acquired in a stepwise fashion. In the case of single-gene hereditary colorectal cancer syndromes, all DNA-containing cells have a germline mutation in one allele of the involved gene and thus are a step closer to the accumulation of additional acquired mutations necessary to lead to CRC.

In human CRC, 80% of the tumors are micro-satellite stable, which means they have intact DNA MMR and can correct single-base and small-loop base-pair mismatches present throughout the non-coding and coding regions of the genome. The remaining 20% of CRC tumors exhibit MSI due to defects in this DNA MMR pathway that corrects small base-pair mistakes in mononucleotide, dinucleotide, and trinucleotide repeat regions throughout the genome and are classified as having high or low MSI (MSI-H or MSI-L, respectively).^5^ A small fraction of MSI-H tumors result from germline mutations in 1 of 4 DNA MMR genes—*MLH1*, *MSH2*, *MSH6,* and *PMS2*—and result in the hereditary CRC condition called Lynch syndrome. However, the greater proportion of MSI-H tumors arises via impairment of DNA MMR through hypermethylation of the *MLH1* gene.

Although only 15% to 20% of sporadic cancers are MSI-H, 90% of patients who meet the Amsterdam criteria for Lynch syndrome have MSI-H CRC.^8^ Tumor DNA can be evaluated for MSI using polymerase chain reaction to amplify a panel of DNA sequences with nucleotide repeats.

Lynch syndrome, an autosomal dominant disorder, is the most common hereditary colon cancer syndrome. Mutations in 1 of the MMR genes usually result in truncated or lost protein product. Thus, tumors can be screened for defective DNA MMR by using polymerase chain reaction to test for MSI or IHC to test for loss of MMR protein expression. The results of IHC may then be used to direct germline sequencing toward a specific DNA MMR gene in young-onset cases or in persons with clinical or family history criteria suggestive of Lynch syndrome. Hypermethylation assays and *BRAF* V600E mutation testing of tumor DNA can be used to distinguish an MSI-H tumor with absent *MLH1* expression caused by hypermethylation of the *MLH1* promoter from a tumor caused by a germ-line *MLH1* mutation. Tumors with hypermethylation of *MLH1* and with the *BRAF* V600E mutation nearly always represent sporadic CRC not caused by a germline *MLH1* mutation and not associated with Lynch syndrome.

In the case presented here, the patient was tested for a familial syndrome because of his relatively young age at presentation (44 years); however, he lacked many of the features of either FAP or Lynch syndrome. The genetic diagnosis of Lynch syndrome requires a germline mutation in 1 of the MMR genes. The patient’s tumor showed normal expression of the MMR genes *MLH1*, *MSH2*, and *MSH6* by IHC. MSI testing was not performed. A previous study from our institution showed that a normal IHC test for MLH1 and MSH2 has a 96.7% positive predictive value for a microsatellite stable/MSI-L phenotype.^9^ On the basis of the IHC data alone, it is highly unlikely that the patient has a germline mutation in an MMR gene, which would lead to Lynch syndrome with MSI.^9^

Another hereditary CRC syndrome that can be considered is *MYH*-associated polyposis (MAP). MAP has a phenotypic overlap with FAP, attenuated FAP, and Lynch syndrome; biallelic carriers have an 80% cumulative lifetime risk of CRC by age 70 years.^10^ In several studies, among patients with early-onset CRC (diagnosed before age 50 years) who tested negative for Lynch syndrome, 1% to 2% were biallelic carriers of the *MYH* mutation.^11^–^13^

## CRC screening and testing recommendations

Although CRC does not usually develop until age 65 years or older, up to 20% of CRC cases will arise in persons 50 years or younger who do not have either of the known hereditary CRC conditions.^1^–^3^,^6^,^14^ The American Gastroenterological Association has published guidelines for CRC screening for average-risk and higher-risk patients.^15^,^16^ Persons with a family history of CRC or adenomatous polyps (a first-degree relative with CRC or adenomatous polyps diagnosed before age 60 years, or 2 first-degree relatives with CRC diagnosed at any age) should have screening colonoscopy starting at age 40 years, or 10 years younger than the earliest diagnosis, whichever comes first, with repeat colonoscopy every 5 years.

If testing for MMR is negative, patients with early-onset CRC may be tested for *MYH* mutations, regardless of their family history or the number of colon polyps. IHC can be used in clinical practice to test for MAP regardless of the specific *MYH* mutations.^17^

## Conclusions

The case presented here highlights that CRC can occur at an age younger than the cancer-screening guidelines suggest for average-risk patients and also shows the importance of using family history to determine the timing of the first CRC screening. Had our patient undergone his first screening colonoscopy at age 40 years as recommended by the American Gastroenterological Association—given that the patient’s father had colon polyps removed before age 60 years—his CRC might have been diagnosed at an earlier stage. Similarly, the patient’s 2-year history of hematochezia warranted a colon examination. We stress the importance of acknowledging and pursuing these symptoms, even in younger patients.
